# The Phenomenology and Neurobiology of Visual Distortions and Hallucinations in Schizophrenia: An Update

**DOI:** 10.3389/fpsyt.2021.684720

**Published:** 2021-06-11

**Authors:** Steven M. Silverstein, Adriann Lai

**Affiliations:** ^1^Department of Psychiatry, University of Rochester Medical Center, Rochester, NY, United States; ^2^Department of Neuroscience, University of Rochester Medical Center, Rochester, NY, United States; ^3^Department of Ophthalmology, University of Rochester Medical Center, Rochester, NY, United States; ^4^Center for Visual Science, University of Rochester Medical Center, Rochester, NY, United States

**Keywords:** visual hallucinations, visual distortions, psychosis, schizophrenia, retina, mechanisms, self

## Abstract

Schizophrenia is characterized by visual distortions in ~60% of cases, and visual hallucinations (VH) in ~25–50% of cases, depending on the sample. These symptoms have received relatively little attention in the literature, perhaps due to the higher rate of auditory vs. visual hallucinations in psychotic disorders, which is the reverse of what is found in other neuropsychiatric conditions. Given the clinical significance of these perceptual disturbances, our aim is to help address this gap by updating and expanding upon prior reviews. Specifically, we: (1) present findings on the nature and frequency of VH and distortions in schizophrenia; (2) review proposed syndromes of VH in neuro-ophthalmology and neuropsychiatry, and discuss the extent to which these characterize VH in schizophrenia; (3) review potential cortical mechanisms of VH in schizophrenia; (4) review retinal changes that could contribute to VH in schizophrenia; (5) discuss relationships between findings from laboratory measures of visual processing and VH in schizophrenia; and (6) integrate findings across biological and psychological levels to propose an updated model of VH mechanisms, including how their content is determined, and how they may reflect vulnerabilities in the maintenance of a sense of self. In particular, we emphasize the potential role of alterations at multiple points in the visual pathway, including the retina, the roles of multiple neurotransmitters, and the role of a combination of disinhibited default mode network activity and enhanced state-related apical/contextual drive in determining the onset and content of VH. In short, our goal is to cast a fresh light on the under-studied symptoms of VH and visual distortions in schizophrenia for the purposes of informing future work on mechanisms and the development of targeted therapeutic interventions.

## Introduction

Hallucinations are one of the characteristic symptoms of schizophrenia and other psychotic disorders, occurring at some point in ~60-80% of those affected (1–3). Although hallucinations in psychotic disorders occur most commonly in the auditory modality, a recent review of visual hallucinations (VH) in schizophrenia reported a weighted mean prevalence of 27%, with a notably wide range across studies (range: 4–65%; SD = 9) (4). A subsequent large-scale study of non-affective psychosis (*N* = 1,119) indicated that 37% of people with schizophrenia and 47.5% of people with schizoaffective disorder had experienced VH at some point (5). Findings from other individual studies suggest that the variability in prevalence across investigations may be due to methodological differences in how VH are assessed, as well as clinical differences across samples. For example, Bracha et al. (6) found that a chart review of discharged schizophrenia patients with a chronic course of illness identified a history of VH in 32% of the cases, whereas their prospective evaluation with another patient sample indicated a VH prevalence of 56%; notably, in 43% of the cases with a history of VH, it was the research interview that first documented this symptom (6). These results suggest that studies that rely on clinician reports may yield underestimates of VH prevalence. Several other studies of people with a chronic course of schizophrenia have reported similar rates, such as 40% (7), 49% (8), 57.2% (in the United States vs. 39% in India) (9), and 63% (10)^1^. These data suggest that, at least in some subgroups of individuals with schizophrenia, the rate of VH may be higher than traditionally thought, and that standard assessments in clinical settings may often fail to adequately probe this type of symptom.

Visual hallucinations are not the only form of visual perceptual anomaly experienced in schizophrenia. For example, over 60% of people with schizophrenia experience visual distortions involving changes in clarity, form, brightness, color, motion, or persistence of visual stimuli (8, 12–18) (see Table 1). It has also been reported that visual imagery is increased in people with schizophrenia (19). In addition to visual anomalies, it is well-established that schizophrenia is associated with visual processing impairments on laboratory tasks, including in contrast sensitivity and perceptual organization (17) (see Table 2). Alterations in such visual functions have also been observed among those at high risk for psychosis (20, 21), and visual perceptual disturbances in childhood (22, 23) and adulthood (24) predict the later development of schizophrenia [and to a greater extent than other sensory anomalies (23)].

**Table 1:** Examples of visual distortions experienced by people with schizophrenia.

**Phenomenon**	**Description**
Blurred vision	Transiently reduced visual acuity
Transitory blindness	Temporary loss of most/all vision
Partial seeing	Parts of objects that should be visible are not visible
Hypersensitivity to light	Brightness and colors appear to be intensified
Photopsias	Light stimuli appear and disappear quickly
Porropsia	Distance between self and objects is altered
Micropsia	Objects seem smaller and/or space larger
Macropsia	Objects seem larger than usual
Metamorphopsia	Objects appear to change in shape and form
Prosometamorphopsia	Faces change and can look disfigured
Metachromopsia	The normal coloring of objects is altered
Dysmegalopsia	Different parts of objects change differently in size
Pseudomovement of objects	Stable objects and scenes appear to move
Changes in object orientation	Upright objects may appear tilted
Palinopsia	Repeated perception of objects that are no longer in the field of view

*For further detail on these phenomena in schizophrenia see Keane et al. (8), Bunney et al. (12), Silverstein (17), Klosterkotter et al. (24), Silverstein et al. (18), Nikitova et al. (25), Keri et al. (26), and Kiss et al. (27)*.

**Table 2:** Definitions and examples of different levels of visual processing, emphasizing those that are clearly impaired in many people with schizophrenia.

**Level**	**Definition**	**Examples**
Low	Representation of information generated by the retina and primary visual cortex	Sensitivity to luminance changes (as measured by electroretinography)^*^ Contrast sensitivity^*^ Edge detection Spatial frequency processing^*^ Orientation tuning^*^ Vernier acuity^*^ Motion detection
Mid	Integration of information about visual features into higher order representations with emergent features	Figure-ground segregation Perceptual organization^*^ Coherent motion detection^*^ Illusory contour perception^*^ Visual context processing (e.g., Ebbinghaus illusion)^*^
High	Interpretation of what is seen	Disambiguation based on prior experience (e.g., Mooney faces test)^*^ Object recognition^*^ Object classification Depth inversion illusions^*^

* *Consistent published evidence of impairments in schizophrenia. Some visual processing paradigms on which people with schizophrenia have consistently shown impairments, but that do not fit neatly into the categories of low-, mid-, or high-level vision, or that operate at multiple levels, are not shown (e.g., backward masking deficits). See Silverstein (17) and Silverstein et al. (28) for a comprehensive reviews of these literatures*.

The visual system is the most understood part of human brain, and the most studied area in neuroscience (29). For these and other reasons, it provides an excellent model system for understanding neural function and circuitry, including for generating and testing hypotheses about what may be occurring in other systems in the brain (30). Therefore, the potential for studies of alterations in visual functions in schizophrenia spectrum disorders to inform our understanding of the development, pathophysiology, and heterogeneity of these conditions appears to be significant. Indeed, there are numerous examples of findings from such studies that have advanced knowledge in these areas, including: (1) the predictive validity of visual disturbances for a later diagnosis of schizophrenia (22–24); (2) the documentation of visual impairments in high risk individuals (20, 21); (3) links between high-level visual changes (see Table 2) and psychotic symptoms (31) suggesting the utility of specific visual tasks for probing predictive coding mechanisms (i.e., the degree to which perception can be considered optimal from a Bayesian perspective, with an appropriate balance of emphases on sensory data and prior experience) (32–34); (4) links between mid-level visual processing alterations and disorganized symptoms suggesting shared impairments at the level of apical dendrite-mediated context-based amplification of feedforward input (17, 35–37) (5) links between low-level visual disturbances and negative symptoms suggesting shared mechanisms involving gain control (38–41); and (6) consistent findings of associations between specific visual impairments and characteristics such as poor premorbid social functioning and poor prognosis (17). It is curious, however, that despite consistent accumulation of information on visual impairments in schizophrenia, *these findings have rarely been applied to an understanding of visual hallucinations and distortions*. One purpose of this review is to address this gap in the literature.

## VH in Schizophrenia

VH in psychosis are typically experienced as being as real and vivid as typical percepts of stimuli in external space, and are generally out of the control of the individual experiencing them, although some people have insight as to the hallucinatory nature of the percepts (4, 42, 43). Content is typically in color (but can be in black and white), complex rather than simple, and includes people (e.g., strangers, supernatural beings, or loved ones), faces, animals, shadows, or other fully formed objects or beings (4, 42–45). VH in schizophrenia are typically three-dimensional and are incorporated into visual scenes (46). However, in some cases, the VH may be in the form of simple geometric patterns (17, 45). In contrast to some of the findings reported above, Lindal et al. (47) found a lower frequency of VH involving people and a higher frequency of unformed images (e.g., lights) in schizophrenia compared to VH in the general population. This is consistent with the findings of an earlier review (48). It is possible these discrepant findings reflect differences across studies in the relative contributions of factors related to the expression of VH with which samples of patients are heterogeneous [e.g., reduced retinal signaling, hippocampal changes, excessive striatal dopamine (DA); see **Figure 2**]. These factors will be discussed in later sections of the paper.

In a study involving patients with various psychotic disorders, including schizophrenia (49), most VH were located in the midline of the visual field or located across both hemifields and appeared to be close to the viewer, but not close enough to touch. One study reported that the majority of VH occurred when patients were alone, in quiet environments, or in dim lighting conditions (49), whereas an older pre-DSM-III study reported that only 6% of people with schizophrenia had VH when they were alone (43). VH may be fleeting or persist for hours at a time (3, 42, 43). The reactions to such visions can be negative or positive, but VH in people with schizophrenia most commonly cause distress, which often leads to functional impairment (4, 42, 49–52). People who have VH as well as hallucinations in other modalities are more likely to report their VH as real, irritating, and distressing. If the content of VH is accepted as real, or possibly real, they can contribute to the formation of delusions (e.g., of control, thought insertion, etc.), which, in turn, can lead to even greater distress (53) and functional impairment. Even the milder changes that characterize visual distortions can be clinically significant. For example, a study of visual distortions in adolescents at a clinical high risk clinic (54) indicated that they were significantly associated with suicidal ideation (OR = 4.33, 95% CI = 1.28–14.64) even after controlling for age, gender, depression, thought disorder, paranoia, and auditory distortions.

Few schizophrenia patients who experience VH do so without also experiencing auditory hallucinations, either simultaneously or at other times. The rate of patients who experience VH and auditory hallucinations has been estimated at 80–98%, whereas it is far less common for those who have auditory hallucinations to also experience VH (1, 3, 6, 7). Of note, the predominance of auditory hallucinations relative to VH in psychotic disorders contrasts to what is observed in neurological disorders such as Parkinson's disease and various forms of dementia; in these latter conditions, VH are much more common than auditory hallucinations (4). The common presence of auditory hallucinations with VH in schizophrenia suggests that aspects of VH pathophysiology in psychotic disorders are shared with that of auditory hallucinations. In the section on cortical mechanisms below we discuss evidence that all forms of hallucinations may share a specific brain network abnormality, while hallucinations in a given sensory modality involve additional impairments specific to that modality.

There are mixed results regarding a possible relationship between VH and illness severity in schizophrenia, although numerous reports suggest that the presence of VH is associated with a more severe course of illness (1, 7). Further evidence for a link between illness severity and VH comes from studies indicating that VH are associated with lower IQ and earlier age of onset (5, 6). It has also been observed that childhood-onset schizophrenia, which is generally characterized by poor long-term outcomes (55–57), is associated with higher rates of VH than is adult-onset schizophrenia (58).

In contrast to these findings, however, are the results from several studies suggesting that VH may be associated with a better prognosis. For example, Small et al. (52) reported that patients with acute schizophrenia who experienced multimodal auditory-visual hallucinations had shorter and fewer previous hospitalizations compared to patients with schizophrenia who did not experience VH Similarly, McCabe et al. (59) found that participants with schizophrenia who were classified as having a “good prognosis” were more likely to have a history of VH than those with a “poor prognosis” profile. Relatedly, a study of young people at clinical high risk for psychosis reported that those with visual perceptual anomalies were at lower risk for conversion to full psychosis than those with auditory perceptual abnormalities (60).

One potential explanation for these discrepant findings regarding VH/distortions and illness severity in schizophrenia is that hallucinatory experiences, including VH, are often associated with a history of severe trauma and post-traumatic stress disorder (61). The clinical presentation of PTSD with VH can be difficult to discriminate from that of schizophrenia in the short term, and these disorders can also co-occur, complicating the diagnostic assessment (61, 62). A history of trauma also increases risk for both PTSD and a psychotic disorder (63, 64). Mood disorders with psychotic features, especially bipolar disorder, can also be difficult to distinguish from schizophrenia, especially early in the illness, and hallucinations in bipolar disorder are more likely to be in the visual modality compared to those in schizophrenia (65). The studies by Small et al. and McCabe et al., which suggested that VH in schizophrenia are associated with a better prognosis, were conducted before the DSM-III era (i.e., prior to the establishment of research-grade diagnostic criteria); thus, these samples may have included people with psychotic symptoms but who would not meet current diagnostic criteria for schizophrenia (e.g., people with PTSD or a mood disorder with psychotic features). This is consistent with the recognition that prior to DSM-III, at least in the United States, the diagnostic category of schizophrenia had become so broad as to include conditions that are no longer considered schizophrenia spectrum disorders, and was applied to many people who would now be considered to have bipolar disorder (66). Evidence for this in the McCabe et al. (59) study is that the good prognosis group (the one with a higher rate of VH) was more likely to have diagnosable major depression or mania, and so it is possible that many of these patients would, currently, be considered to have a mood disorder with psychotic features rather than schizophrenia. With regard to the more recent study of individuals at clinical high risk for psychosis (60), although speculative, it is possible that young people presenting at the high-risk clinic with visual perceptual disturbances were more likely to be characterized by PTSD or by what would eventually turn out to be a mood disorder compared to those without such symptoms; if this were the case, it may have contributed to the reduced risk of conversion to a psychotic disorder among those with visual perceptual symptoms in that sample. In short, while VH is not, in general, a good prognostic indicator when compared to not having any hallucinations, when VH occur early in a psychiatric illness it may sometimes be an aspect of severe PTSD or a mood disorder in people who are not likely to develop a schizophrenia spectrum disorder. In contrast, in people with a definite diagnosis of a schizophrenia spectrum disorder, especially those with a history of chronic illness, the occurrence of VH appears to be an aspect of a more severe psychotic disorder. It is interesting that very poor prognosis schizophrenia patients are characterized by gray and white matter loss in the occipital lobe (67, 68), whereas this is more rare in patients with better outcomes. It is unclear, however, if such volume loss in visual cortex is related to VH or visual distortions in this patient group.

## Syndromes of Visual Hallucinations

ffytche (69) proposed that there are three independent visual hallucinatory syndromes, each of which have their own patterns of pathophysiology, content, and other associated phenomena. One, the *deafferentation syndrome*, involves loss of input to the visual cortex due to retinal and/or subcortical impairment. It encompasses visual anomalies related to eye conditions such as macular degeneration, Stargardt's disease, and glaucoma. The VH associated with the deafferentation syndrome can be simple, geometric patterns or fully formed objects and people. The latter case is known as Charles Bonnet Syndrome. With regard to mechanisms, it is thought that the reduction in, or complete loss of, visual input associated with these eye conditions disinhibits visual cortical neurons, which results in a release of normally dormant patterns of activation that are subjectively experienced as VH (70). This model overlaps with the Activation, Input and Modulation (AIM) model proposed by Hobson et al. (71), which was applied to VH in Parkinson's disease and Lewy body dementia by Diederich et al. (72). It has been shown that such an increase in excitability consequent to loss of retinal input in part reflects increased plasticity and cortical reorganization following a lesion. For example, animal and computational studies indicate that after a visual cortical lesion, peri-lesion neurons increase their receptive field size, and their activity level is increased due to removal of inhibitory input from the lesioned neurons (73, 74). This activity has been demonstrated in cat to result in illusions and perceptual filling-in phenomena (75). Hyper-excitability of visual circuits has been associated with stimulation-induced VH in healthy volunteers (76), and with spontaneous VH in Charles Bonnet syndrome (77). A good summary of data on hyper-excitability after reduced input to the visual system, and its relationship to VH can be found in Burke (78). Data on visual system hyper-excitability and VH in schizophrenia, Parkinson's disease, and LSD use is reviewed in Waters et al. (4). It must be noted, however, that while retinal/LGN deafferentation typically leads to hyper-excitability in visual cortex, lesions in V1 or V2 can also lead to disinhibition of activity within the occipital lobe that can be associated with VH (79). Hyper-excitability, in the form of seizure-like activity [“local paroxysms in the sensory system” (78), p. 537] can, in general, be a result of isolation of cortical circuitry due to brain trauma, lesions, hypoxia, or other causes. While this can be thought of as a form of deafferentation, it is not strictly the type of sensory deafferentation that ffytche discussed in his 3-syndrome model.

In the typology proposed by ffytche (69) [see also (76)], schizophrenia was not included in the group of conditions characterized by the deafferentation syndrome. There and in a later paper (76), he concluded that sensory deafferentation-induced simple hallucinations such as those found in Charles Bonnet syndrome are not a good model for VH in disorders such as schizophrenia where the early visual system is thought to be intact, and VH are thought to involve reduced cholinergic inputs from the basal forebrain and subsequent effects on ventral temporal regions (resulting in complex VH; see paragraph below). However, as we describe in a later section, in the past decade and especially within the past 5 years, there has been consistent reporting of retinal functional and structural impairment in schizophrenia, specifically in the form of reduced strength of retinal neural cell output and thinning of retinal neural layers (28, 46, 80). In addition, some of the visual distortions reported by people with schizophrenia resemble those seen in cases of retinal disease (18, 24, 25). There is also emerging evidence that glaucoma is a neurodegenerative condition whose occurrence precedes a range of neurodegenerative disorders [e.g., Alzheimer's disease (81) and mild cognitive impairment (82), as well as indirect evidence for glaucoma-like changes in schizophrenia, including an enlarged cup-to-disc ratio (83) and abnormalities in frequency doubling perimetry reviewed in Almonte et al. (84)]. Recent findings also indicate that glaucoma often precedes a diagnosis of schizophrenia, bipolar disorder, or major depression by several years (85). These observations converge to suggest that some of the abnormal visual activity in schizophrenia may be secondary to reduced retinal input to the visual cortex, a hypothesis that received preliminary support in a computational model (86). An important distinction, however, is that while people with Charles Bonnet syndrome have insight into the non-reality of their VH in most cases, in schizophrenia the VH are likely to be experienced as being part of a changed reality.

The second syndrome described by ffytche (69), the *cholinergic (Ach) syndrome*, is caused by dysfunction in the brainstem and ascending brainstem neurotransmitter pathways, particularly the projections of the cholinergic pathway to the basal forebrain and cerebral cortex (87). This syndrome is defined by illusions, delusions, and fully formed, hallucinations in multiple modalities, and ffytche suggested that it may underlie the psychotic symptoms associated with neurodegenerative disorders such as Alzheimer's disease, Parkinson's disease, and Lewy body dementia. In terms of the three syndromes described by ffytche, Waters et al. (4) suggested that the Ach syndrome is the best fit for schizophrenia from a phenomenological perspective, with the caveat that there are important differences between schizophrenia and the other conditions listed in the category (see below). Evidence in support of the view that Ach is involved in VH in schizophrenia is that the disorder has been characterized by reduced Ach transmission, and a reduction in both muscarinic and nicotinic Ach receptors (88). Deficient cholinergic transmission at the level of the basal forebrain leads to reduced signal-to-noise ratios (89, 90), and therefore to an increased reliance on stored information in determining the nature of incoming stimuli, whereas basal forebrain Ach activity decreases effects of top-down projections and increases reliance on sensory data (91). Deficient cholinergic transmission may, therefore, be a mechanism involved in hallucination formation in schizophrenia and other conditions, as described by the predictive coding model of psychosis (92). Further evidence for the Ach hypothesis of VH in schizophrenia comes from findings that high doses of anticholinergic medications can induce a psychotic state [reviewed in Terry (88)], that normal doses of these medications can induce recurrence of psychosis (93), and that acetylcholinesterase inhibitors (which increase the amount of acetylcholine available in the synaptic cleft), used in conjunction with antipsychotic medication, can reduce the frequency of VH (94, 95). In addition, a recent model of VH in synucleinopathies (e.g., Parkinson's disease, Lewy Body dementia, and multiple system atrophy) posits that they result from loss of suppression of the default mode network (DMN) by task-positive networks during wakefulness, and that this is a consequence of cholinergic dysfunction (96–102). Inadequate suppression of DMN activity is thought to lead to the release of unconscious representations that can be considered priors in the emerging altered or dissociative state, which then supersede priors related to current external stimuli. This proposal is essentially a predictive coding model. The biological basis of the proposed disturbance is thought to involve alterations in DA signaling, as well as in serotonin and Ach activity, all of which are highly interactive (103, 104). Further consideration of this network model, as it may apply to schizophrenia, is found in a later section of the paper on cortical mechanisms. In contrast to the hypothesis of Ach involvement in VH in schizophrenia, Waters et al. (4) noted that the clinical presentation of hallucinations in schizophrenia differs from what is observed in the other disorders in this category in terms of age of onset, emotional content, attributions of their origins (i.e., level of insight into their reality in external space), fMRI findings regarding potential mechanisms, and the predominance of auditory over visual hallucinations. Therefore, while cholinergic dysfunction may contribute in part to VH in schizophrenia, additional research is needed to clarify its interaction with other factors that determine that nature of VH in people with the disorder.

The *serotonergic (5-HT) syndrome* is the third syndrome identified by ffytche (69). It is typically characterized by simple VH that are thought to be due to an overactive serotonergic system. Examples of this syndrome include LSD flashbacks, migraine auras, and MDMA-induced hallucinations. Although neither ffytche (69) nor Waters et al. (4) include schizophrenia in this group, and while schizophrenia does not seem to be primarily a disorder of altered serotonergic transmission, findings from multiple lines of research implicate serotonergic dysfunction in this condition (105, 106). For example, based on several observations, including serotonergic effects of hallucinatory drugs such as LSD, the modulatory role of serotonin on DA transmission, and the substantial antagonistic action of the atypical antipsychotic medication clozapine at serotonin 2A (5-HT2A) receptors relative to its modest affinity for D2 receptors, it has been suggested that increased activity at the 5-HT2A receptor may be related to the positive symptoms in schizophrenia (103, 105, 107–110). On the other hand, a recent review of emerging 5-HT2A receptor antagonists for the treatment of schizophrenia (pimavanserin, roluperidone, lumateperone, ritanserin, and volinanserin) suggested that these drugs have their strongest effects on negative and cognitive symptoms, and that their antipsychotic effects involve reducing the likelihood of DA receptors transitioning to a supersensitive state in response to other (DA receptor blocking) antipsychotic medications (111). In short, despite substantial research in this area, the direct and indirect roles that serotonergic alterations play in the pathophysiology of SZ are not yet clear. With regard to VH in psychotic disorders specifically, it has been noted that the differences in the nature of hallucinations in schizophrenia and in altered states induced by hallucinogenic drugs [reviewed in Leptourgos et al. (112)] suggest that VH in schizophrenia are not primarily the result of excessive serotonergic tone.

In addition to the three VH syndromes discussed above, in a subsequent paper ffytche (76) considered the issue of topological (i.e., region-specific) vs. hodological (i.e., network connectivity) changes related to VH. An integration of these views led to the *hodotopic framework*, in which VH are seen as due to both hyper-excitability in specific cortical regions, as well as hypo-connectivity and/or hyper-connectivity between visual cortex and other brain regions. Both Burke (78) and ffytche (76) suggested that while VH content is largely defined by the region undergoing excess activation (e.g., fusiform face area in the case of faces, V1-V2 for simple features and geometric patterns), it is the deficient network activity that is necessary for VH to occur (see section on cortical mechanisms below for more on this). An additional relevant set of dimensions was proposed by Oertel et al. (113), who suggested that excess hippocampal activation determines the extent and nature of memory-related contents in VH, whereas activation in sensory regions may determine the vividness of the VH (see section on cortical mechanisms, for more detail on hippocampal involvement in VH in schizophrenia).

The hodotopic framework, alongside the preliminary evidence for involvement of the hippocampus in VH in schizophrenia (see next section), is consistent with the view we propose below in the sense that changes in network function occur alongside regional changes, and that life experience and stored representations can be understood as driving individual differences in the extent of memory fragment-related imagery in VH, and therefore in the nature of and emotional reactions to VH. However, as we discuss below in the section on the retina, evidence has been accumulating that suggests direct and indirect effects of retinal impairment on VH in schizophrenia, and therefore that the assumption of a lack of involvement of low-level sensory impairment in VH in schizophrenia was premature.

## Cortical Mechanisms of VH and Visual Distortions in Schizophrenia

As noted above, theories of VH implicate reduced visual input and subsequent cortical compensations; cholinergic dysfunction leading to low signal-noise ratio and the subsequent excessive influence of top-down priors (experience-based hypotheses) during the generation of perceptual representations; and/or serotonergic dysfunction. Recent models of VH have focused more heavily on network dysfunction, although this was implied in some of the older theories, and explicit in models involving disinhibition of the DMN. Evidence for involvement of network-level dysfunction in VH in schizophrenia comes from a resting-state fMRI study by Ford et al. (114), which demonstrated that patients with VH show hyperconnectivity between the amygdala and cortical areas involved in higher order visual processing. This link may explain the emotionally intense content and often distressing reactions to VH in people with schizophrenia. As noted above, involvement of regions of the hippocampus may determine the extent of memory fragment inclusion in VH in schizophrenia. A resting-state fMRI study demonstrated increased connectivity between the nucleus accumbens and the insula, putamen, parahippocampal gyri, and ventral tegmental area in patients with VH compared to patients with only auditory hallucinations (115). Further evidence suggesting both local and network-related aspects of hippocampal dysfunction has been found in multiple studies, including findings that both hypertrophy of the hippocampus, and hyper-connectivity of this region with frontal and visual regions is found in schizophrenia patients with VH compared to patients with auditory hallucinations alone (116). Other evidence of dysregulated hippocampal activity and its role in network changes associated with VH was observed by Hare et al. (117) and Amad et al. (116). The role of hippocampal abnormalities leading to altered relational memory links, and their role in VH was discussed by Behrendt (118). Finally, neurodevelopmental evidence consistent with hippocampal involvement in VH comes from findings of incomplete hippocampal inversion in schizophrenia patients with VH compared to those with only auditory hallucinations (119). While Jardri et al. (120) did not find evidence of abnormal hippocampal/parahippocampal gyrus activation in hallucinating schizophrenia patients, they nevertheless suggested that instability in firing from this area could “trigger dispersed storage sites within modality-dependent association sensory cortices, thereby causing hallucinations” (p. 1114). Of note, increased input of memory-related material into consciousness is consistent with findings of disinhibition of DMN activity during waking consciousness, given the role of the hippocampus and parahippocampus in the medial temporal subsystem of the DMN.

A recent study examined network components that are common to hallucinations in general, and components that are specific to the sensory modality of a hallucination (121). In this study of patients with focal brain lesions, 90% of focal lesions occurring in people who subsequently developed hallucinations had positive connectivity to the cerebellar vermis lobule VI, positive connectivity to bilateral cerebellar lobule X, and negative connectivity to the right superior temporal sulcus. In patients with VH, 95% of lesions demonstrated connectivity to the LGN. Interestingly, 95% of subcortical lesions associated with VH demonstrated negative connectivity to extrastriate visual cortex (Brodmann areas 18 and 19), while 100% of cortical lesions associated with VH demonstrated positive connectivity to this same region. Moreover, in 60 of 61 cases, the subcortical or cortical region associated with VH demonstrated significant connectivity to the *same* region in extrastriate visual cortex. Although people with prior histories of neurologic or psychiatric disorders (including schizophrenia) were not included in the Kim et al. study, the data suggest that involvement of cortical and subcortical visual regions are a necessary component for the experience of VH, whereas a wider network may be involved in enabling the shift away from a state in which external stimuli determine what is perceived as external.

Other findings related to the issue of general vs. modality-specific aspects of hallucinations come from a study by Jardri et al. (120), in which hallucinations were associated with unstable DMN and task network dynamics, involving transient disengagement of the DMN—as occurs with external stimulation, but in this case in the absence of anything external that should lead to the (hallucinated) percept. This abnormal and unexpected transitioning between resting and task-positive states was found to be a general correlate of hallucinations, independent of sensory modality. This study also found that increased activity in primary sensory cortices was not necessarily associated with hallucinations, but that activity in association sensory cortices was related specifically to the sensory modality of the experienced hallucination. Of note is that the findings of Jardri et al. (120) on disengagement of the DMN differ from those cited earlier in which disinhibition of DMN activity was associated with VH. This difference may be due to studying brain activity during VH (120) in contrast to studying characteristics associated with having VH in general (i.e., trait-level setting conditions). Evidence consistent with this view comes from an fMRI study of auditory hallucinations in which changes in network connectivity over time were not associated with severity of hallucinations on the day of scanning, but patients more likely to hallucinate in general showed a weaker inverse relationship between the DMN and task-positive networks than patients with a lower general frequency of hallucinations (122). On the other hand, Jardri et al. studied people with brief psychotic disorder, whereas studies that have focused on VH and DMN activity were conducted in neurological populations (although studies linking increased hippocampal connectivity with VH in schizophrenia are relevant to the disinhibition of DMN hypothesis (see above), as are findings of ketamine-induced reduced suppression of DMN activity [e.g., (123)] and auditory and visual hallucinations and distortions (124). See below, the section on an updated model of VH in schizophrenia, for further discussion of these issues.

**Figure 1:**
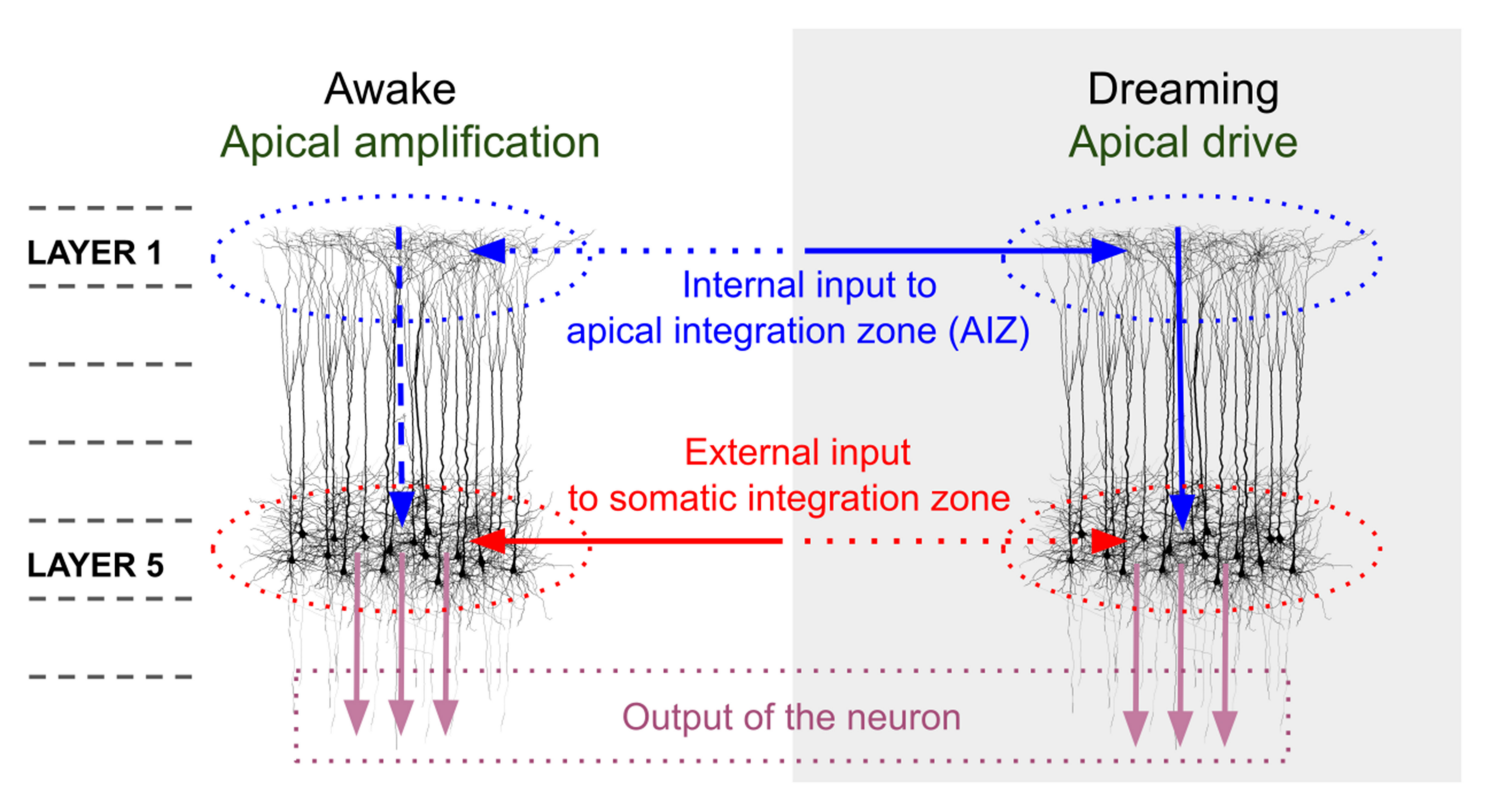
Comparison of pyramidal cell component contributions during waking and dreaming consciousness. The image on the left demonstrates that during waking consciousness the primary determinants of perception are external inputs (continuous red arrow), which are processed as feedforward activity through the somatic integration zone (red dotted ovals) of layer 5 pyramidal neurons. This activity can be amplified or suppressed based on internal contextual input (including episodic memory traces, expectations, and emotional factors; horizontal blue dotted arrow), which is processed in layer 1 of the same neurons, via activation of apical dendritic tufts. The combined contextual activity is integrated within the apical integration zone (AIZ) of pyramidal neurons (blue dotted ovals), whose activation level affects the firing rate of the neuron (vertical blue dotted arrow), but does not lead the neuron to fire in the absence of external input. During dreaming, internal contextual input (continuous horizontal blue arrow) can activate an apical dendritic mechanism that enables it to drive the neuron's output (continuous vertical blue arrow), and that output is interpreted (by downstream circuits) as conveying information about the external world (external input) even though it does not. We propose that in the case of visual hallucinations a scenario midway between the two extremes of waking consciousness and dreaming is operative, wherein AIZ activity can exert both modulatory *and* driving influences, resulting in internally-generated visual representations, often involving significant contributions from trauma-based memories and related emotional and symbolic material, being experienced as having been externally generated. In the view proposed in this paper, complex hallucinations involving this mechanism differ in many respects from simple visual hallucinations and visual distortions in that the latter two phenomena primarily represent compensations within the visual system for missing, weakened, or degraded input, and with relatively fewer contributions from episodic memory and emotional factors. However, even simple VH can be related to as meaningful and can reflect psychological factors (15). The image in this figure, and portions of the figure caption, are reproduced from: Aru et al. (126). Apical drive-A cellular mechanism of dreaming? *Neuroscience and Biobehavioral Reviews* 119, 440–55, with permission from Elsevier via a CC BY license.

In addition to network mechanisms, a cellular mechanism that may be important in understanding VH in schizophrenia involves disturbances in the influence of apical dendritic activity on the somatic compartment of pyramidal neurons. It has been proposed that during normal waking consciousness, information about external events is processed via feedforward mechanisms involving the somatic compartments of pyramidal cells in cortical layer 5, while this activity is modulated by activity from areas outside the classical receptive field, and more distant regions of the brain (representing contextual, mnemonic, and other “top-down” influences), via synapses onto apical dendrites of pyramidal cells in superficial cell layers (e.g., layer 1) (35, 36, 125). Activity from different inputs to the apical dendrite are integrated in the apical integration zone (AIZ), prior to AIZ output affecting the cell's firing rate or timing. During sleep or under anesthesia, arousal level and degree of external input are greatly reduced, and so there is proportionally greater activation at apical relative to soma dendrites (see Figure 1). This allows the combined AIZ activity to drive (as opposed to only modulate) activity in pyramidal neurons (36). A result of this can be processing of AIZ activity as if it were external stimulation, rather than activity that normally serves only to alter the strength or timing of firing, as in the waking state (126). This effect, called *apical drive*, has been proposed as a mechanism involved in dreams, but also in mental imagery (125, 127–130), and alterations in this mechanism could be involved in the formation of VH and also (at a milder level) visual distortions, as we delineate further below. A detailed account of how disruptions in apical amplification could account for a range of features of schizophrenia can be found in Phillips et al. (35). Similar to the predictive coding and excessive DMN activity hypotheses, this proposal suggests that there is an excessive influence of stored information relative to external input in determining perceptual experience. The hypothesized neurobiology is different, however, in that it is seen as involving multiple neurotransmitters (e.g., glutamate, DA, Ach), including reduced noradrenergic activity. As discussed further below in the section on an updated model of VH in schizophrenia, the material that serves as context may vary greatly depending on whether a person is engaged in instrumental activity (when task-positive brain networks are active), or is focused on internal states or daydreaming (when DMN activity is relatively increased), or is dreaming during sleep (when DMN activity is further increased, along with other changes in brain function). Along this continuum, it can be expected that the nature of contextual input will become more self-referential, more tied to basic issues around security/danger and fulfillment of biological needs, and more based in symbolism and visual/auditory imagery than on logical relationships (131). This may account for the frequent appearance of religious and other archetypal content in the VH of people with schizophrenia (132, 133).

Evidence in support of increased apical drive in VH in schizophrenia comes from studies indicating that schizophrenia patients with VH are more likely to mis-remember items as pictures that had been presented to them as words (134–136). This suggests that the original memory trace is having a reduced driving effect on the conscious representation, whereas the more distant associations to the word (e.g., pictorial representations) that would normally serve a modulatory role are driving what is remembered. Interestingly, in the Aynsworth et al. (134) study the high VH group also reported more intense visual imagery, and more negative visual imagery, findings that are consistent with hyperconnectivity between the hippocampus and other regions (see above), and with histories of traumatic experience (137, 138) (which itself has been related to hippocampal atrophy secondary to neurotoxic effects of chronically elevated cortisol).

Finally, it has long been recognized that excessive DA activity is involved in the genesis of positive symptoms [e.g., (139, 140)]. For example, increases in DA turnover in the associative striatum were associated with hallucination severity (all types combined, and so presumably mostly auditory), especially under conditions of uncertainty in people with schizophrenia (141). Other evidence has also linked DA activity with hallucinations (including the combination of auditory and visual hallucinations) in schizophrenia (115, 142). Data from these and other studies fit well with the proposed role of DA in the predictive coding disturbances in psychosis (143), including the view that hallucinatory activity represents excessive influence of priors. Therefore, increased striatal DA may be another mechanism, in addition to reduced cholinergic activity, that tips the balance of processing toward an increased influence of internally generated representations on conscious perception.

The findings reviewed in this section highlight the increasingly appreciated potential roles of cellular, and local and long-range brain network activity in the genesis of VH in schizophrenia. This includes the contributions of stored information, expectations, and other “top-down” inputs, as well as changes in the relative strength of bottom-up signals. Regarding the latter, Bernardin et al. (46) noted that there has been a lack of appreciation of possible retinal contributions to VH in schizophrenia, in contrast to the study of other neuropsychiatric conditions such as Parkinson's disease. In the following section, we provide an updated summary of this literature.

## Potential Retinal Contributions to VH and Visual Distortions in Schizophrenia

An important and largely unaddressed question in the VH and visual distortions literatures in schizophrenia is the extent to which these symptoms may be due to altered retinal structure or function in the disorder. Since the publication of the typology proposed by ffytche (69), and even since the more recent reviews of Waters et al. (4) and Bernardin et al. (46), there has been rapid growth in the field of *oculomics* [i.e., the study of retinal markers of brain and systemic diseases (144)]. This includes many studies demonstrating retinal structural and functional impairment in schizophrenia, and a small number of papers on altered retinal microvasculature. Comprehensive reviews of this literature have been recently published (28, 80, 144), and so only a brief review of the relevant findings are provided here.

There is now convincing data from a number of studies, published in different countries across multiple laboratories, demonstrating that there is thinning of multiple retinal layers in people with schizophrenia (145–151). These layers include the innermost layer, the retinal nerve fiber layer (RNFL), comprising the axons of the ganglion cells, which form the optic nerve that leaves the retina and synapses at the lateral geniculate nucleus (LGN) of the thalamus; the ganglion cell body and inner plexiform layers; and the vascular layers (e.g., choroid) that support the outermost neural layers (28, 80). There is also evidence for other structural retinal abnormalities in schizophrenia, such as an increase in optic cup volume and cup-to-disc ratio (83), suggesting changes similar to those observed in glaucoma (83). These findings raise the possibility that retinal signals reaching the LGN, and eventually V1, are degraded, and that compensatory processes could lead to visual distortions and VH, as described above (46). Evidence for such a pathological compensatory process has been observed in Charles Bonnet syndrome (152). In Parkinson's disease, one study demonstrated a relationship between RNFL thinning and VH (153), but a second study found no evidence for such a link (154). A recent study in schizophrenia (155, 156) indicated that four separate samples of patients with both auditory and visual hallucinations of at least moderate severity were characterized by significant macula and RNFL thinning relative to a control group. The lack of a control group of patients with auditory hallucinations only and/or no hallucinations precludes a definitive conclusion regarding specific relationships with VH, however. Therefore, the issue of retinal structural contributions to VH in schizophrenia needs further clarification.

Alterations in retinal function have also been observed in schizophrenia using flash electroretinography (fERG) and pattern electroretinography (pERG) [reviewed in (28, 80)]. These data indicate weakened and delayed signaling from photoreceptor and bipolar-Muller cells, and weakened signaling from ganglion cells. These studies have generally not investigated relationships between functional retinal impairment and VH. However, a recent study (157) reported that schizophrenia patients with lifetime VH were characterized by slower signaling in bipolar cells and retinal ganglion cells compared to patients without a history of VH. This is consistent with findings of a relationship between ERG latency increases (and amplitude decreases) and VH in Parkinson's disease (158).

An implication of the findings of impaired retinal structure and function in schizophrenia is that the visual cortex can be thought of as characterized by low-level sensory deafferentation. While this is not as severe as in cases of advanced retinal disease, it may be sufficient in many people to cause phenomena such as visual distortions, and simple and complex hallucinations. When combined with the disturbances noted above that are not characteristic of conditions such as for example, Charles Bonnet syndrome, including relatively increased apical drive which can lead to confusion between reality and fantasy, heightened input from limbic regions, and disinhibition of the DMN, these release phenomena may be elaborated upon into more complex and emotionally intense VH. A good example of how a simple VH involved a complex set of meanings is described in Kaminski et al. (15).

A more subtle consequence of altered retinal, LGN, or early visual cortical activity in schizophrenia is that neural representations of visual input may be degraded or otherwise ambiguous. While this would not necessarily lead to release phenomena as traditionally defined, it would create a burden on predictive coding mechanisms by increasing the number of required comparisons between top-down and bottom-up signals (86, 159). This increased computational burden could potentially lead to errors in interpreting the nature and significance of visual stimuli, such as the generation of representations that are based excessively on top-down information (e.g., hallucinations), or that involve combinations of veridical perception and aspects of non-veridical representations (e.g., visual distortions). This view is consistent with the proposal of Collerton et al. (160), which emphasizes the multiple proto-object representations that can be activated by a single stimulus, and the role of attentional processes (often guided by top-down goals and other schemata) in selecting among the competing hypotheses/percepts for allowing access to conscious awareness. According to this view, VH occur when the top-down processes lead to the selection of a perceptual representation that is unrelated to the external stimulus.

Taken together, the few relevant findings suggest that it is reasonable to further investigate the potential role of retinal dysfunction to VH in schizophrenia. Cross-sectional data are needed, as are longitudinal studies that relate changes in retinal structure and function over time to the emergence of visual perceptual symptoms and state- and stage-related changes in these symptoms.

## Associations between Visual Processing Impairments With VH and Visual Distortions in Schizophrenia

As noted, despite the extensive literature on visual processing impairments in schizophrenia, there have been few attempts to investigate potential links between these impairments and visual distortions and/or VH in this condition. We are aware of three studies exploring links between the well-documented visual processing impairments in schizophrenia and visual distortions or VH. Keri et al. reported that, in a sample of people with a history of schizophrenia (mean age = 34.1 years), impairments on low- and mid-level visual processing tasks were related to increased self-reports of visual distortions (26). Kiss et al. (27) reported that, in a sample of first episode patients, increased magnocellular activity, as reflected by enhanced contrast sensitivity for low spatial frequency information, was related to an increase in perceptual distortions in people with schizophrenia. As noted above, a recent study by Bernardin et al. (157) demonstrated a link between anomalies in the ERG, including slower signaling of bipolar and ganglion cells, and visual hallucinations in people with schizophrenia. This evidence suggests that laboratory measures of low- and mid-level vision may be useful in clarifying mechanisms involved in visual distortions and possibly VH. Additional studies are needed to replicate these findings, and to clarify the role of high-level visual disturbances to VH, especially as these have been related to psychotic symptoms in people with schizophrenia (31).

## An Updated Model of VH and Visual Distortions in Schizophrenia

The findings reviewed above suggest that several factors may be involved in VH in schizophrenia, including: alterations in retinal structure and activity, such as delayed, weak, and/or ambiguous signaling, and compensatory, predictive and release processes initiated within visual cortex; impairments in functioning in visual processing regions [e.g., those that lead to the well-documented changes in contrast sensitivity, perceptual organization, motion processing, etc. (17)]; changes in activity in ascending cholinergic pathways; and abnormal network connectivity, including excessive influence of DMN activity and increased apical drive.

A recent comprehensive view of VH that integrates several of these factors, to account for VH in synucleopathies (100), proposed that cholinergic imbalance leads to thalamocortical dysrhythmia, which results in reduced modulation of activity in cortical-striatal-thalamo-cortical loops. One consequence of these alterations is that there is less inhibition of the DMN from task-positive networks such as the dorsal and ventral attention networks. It has been proposed that disinhibition of the DMN causes it to enter into “an unstable, entropic, state that unleashes the production of oneiric, dissociative, or altered state (sic) of consciousness, and ultimately VH” [(98), p. 4] (96, 97, 99, 100). This hypothesis regarding VH mechanisms is consistent with the proposal that disinhibition of DMN activity contributes to psychotic symptoms in schizophrenia (123, 161, 162). Because the DMN is thought to play a critical role in maintenance of the sense of self (163), including integration of autobiographical memories (164), disinhibition and dysregulation of this network would be expected to lead to VH that are self-relevant and that often contain fragments of memories of emotionally intense experiences (e.g., those related to trauma), and other content that frequently serves as basis for rumination (165), including images and ideation associated with religious themes. It is possible that hallucinations in schizophrenia, more so than in synucleopathies, involve an additional contribution of excessive striatal dopamine, which could further shift the balance of bottom-up and top-down activity toward the latter.

An important consideration in considering the role of apical drive in VH is that the nature of what serves as context is likely to be different across mental states, especially between normal task-positive behavior and default mode activity. For example, during a state of highly focused attention and task activity, the nature of contextual input to a given neuron will be different than it would be during daydreaming or dreaming while asleep. During focused activity, contextual inputs should involve memories related to prior experiences with the current task, and ideation and linguistic associations associated with statistical regularities in the world related to task performance (i.e., to outcomes that can realistically occur). During daydreaming or internally focused states associated with anxiety, however, these constraints are likely to be loosened, resulting in more wide-ranging, illogical, and emotion- and fantasy-based associations (166–168). During dreaming while asleep, there is likely to be the greatest proportion of imagery and ideation related to safety and security issues, and other basic biological concerns (e.g., fear, rage, sexual arousal, isolation), with the links between ideas often expressed using non-logical connections based on emotions, symbolism, sounds, colors, and other basic stimulus features. To the extent that the content of hallucinations reflects components of sensory memories that are inappropriately activated by association [as has been proposed with auditory hallucinations (169)], it would be expected that VH will contain imagery from prior memories or symbolically related to those events. In short, VH may involve transient relative increases in the effects of normally non-conscious emotional and ideational activity, and a qualitative shift wherein this mental content is processed as feedforward (i.e., sensory) activity rather than modulatory influences. In addition, the frequent archetypal content of VH in people with psychotic disorders, which includes imagery such as God (or gods), Jesus, the Virgin Mary, the devil, saints, and angels (132, 133), suggests that in many cases the VH imagery is part of a complex of mental representations that involve strong emotions related to the concept of the self [possibly secondary to increased connectivity between the amygdala and cortical regions involved in higher visual processing, as noted above (114)], and fragments of visual memories, that are most productively related to in symbolic form. This combination of influences may explain the numinous or highly compelling nature of visual hallucinations: the images are not only surprising (as would be, for example, an image of a ruler appearing in front of oneself), they also reflect deep-seated emotional, self-maintenance, and survival concerns and are felt to be highly self-relevant. An excellent phenomenological analysis of VH, presented within the context of a predictive coding account, was provided by Kaminski et al. (15). In this case, the VH of a thin veil of rain that fell in front of the patient was interpreted as an aspect of a self-preservation tendency to prevent fusion between the self and the outer world.

In addition to the factors reviewed in the above paragraph, we suggested that the role of the retinal changes in schizophrenia reviewed above needs to be given greater consideration. The co-occurrence of VH and retinal changes in schizophrenia (157) highlights the need to clarify the extent to which altered visual system input contributes to thalamocortical dysrhythmia, or otherwise contributes to or modifies the level of cortico-thalamic connectivity, or the types of outputs associated with DMN dysregulation. Regarding the latter, we suggest that, in schizophrenia, when the quality of representations in the visual system is more degraded and/or ambiguous than normal due to changes in precortical pathways [e.g., in the retina (80) or optic radiations (170, 171)], a condition is created which increases the likelihood of distorted visual perceptions, activation of inappropriate visual representations, and VH. That is, consistent with the predictive coding model, ambiguities with regard to the nature of visual input—-due to poor-quality signals and compensatory responses such as broadening of neural tuning functions (86, 159, 172–175)—-will further increase the influence of priors in the generation of visual representations. Further, within the context of DMN dysregulation, these priors become more likely to involve material related to security and safety concerns and to imagery and symbolism around these, than during task-positive brain states. In cases where there are histories of trauma, bullying, chronic social defeat and other negative experiences, which occur at an elevated frequency in the histories of people with schizophrenia, VH may become especially disturbing (176, 177). This link with memories of threats to the self, and related thoughts and feelings, may account for why hallucinations in schizophrenia are more likely to be perceived as real, and to be more distressing than, for example, those in Parkinson's disease or Charles Bonnet Syndrome.

If the view proposed here is valid, it becomes necessary to explain why many schizophrenia patients do not have any VH, and why among those that do experience VH, the frequency and form they take can vary to the extent that they do. The answers to these questions are not clear at present. One clue comes from data reviewed above that VH are associated with a more severe form of illness [including poorer pre-diagnosis functioning (8)] and that, in schizophrenia they rarely occur in the absence of AH. It may be the case, therefore, that specific genetic, neurodevelopmental, and/or neurodegenerative factors that increase symptomatology and disability over time also lead to changes in the visual system. This is consistent with findings of occipital lobe atrophy in the poorest outcome schizophrenia patients (67, 68), and with preliminary evidence of greater retinal atrophy in more chronically ill patients (148, 178) and those with more positive symptoms (179), although the latter finding has not been consistently replicated [e.g., (149) found relationships between retinal atrophy and negative, but not positive, symptoms]. An important task for future research, therefore, involves clarifying the factors that determine the differential expression of the proposed mechanisms across patients. Specific questions include whether the VH of patients with retinal impairments differ from those with normal retinal function (and if so, in what ways), and exploration of the differences (e.g., in trauma history) between patients with threatening or survival-related VH imagery and patients with VH without such content [geometric hallucinations or VH involving lilliputian figures (180)].

## Additional Questions and Future Directions

Although there are now several likely candidate mechanisms for involvement in VH, there is still much to be learned about VH in schizophrenia. Two critical issues involve causality, and which mechanisms, if any, can be considered necessary and sufficient for VH to occur. The data reviewed above come mainly from studies comparing schizophrenia patients with vs. without VH, or from correlational studies. Causality cannot be determined using these study designs. Studies that directly influenced each of the mechanisms discussed, and candidate mechanisms that emerge in the future, are necessary. For example, brain stimulation, neurofeedback, or pharmacological manipulations that lead to VH, and whose cessation leads to termination of VH, would provide more convincing evidence about causality.

Regarding necessary and sufficient factors—-no single factor has been found to be necessary or sufficient in studies of VH in schizophrenia. As noted above, a network common to hallucinations caused by brain lesions has been identified (121), but it is not clear whether this network is a necessary component of VH in schizophrenia. That study also demonstrated that VH specifically were associated with altered connectivity with the LGN and extrastriate cortex. Importantly, however, VH in cases of brain injury can differ from what is experienced by people with schizophrenia, and regions outside of the network identified by Kim et al. (121) (e.g., amygdala, hippocampus, visual association areas) have been linked to VH in people with schizophrenia, as discussed above. In short, it is not possible to demonstrate at this point that a specific set of brain regions or a network is necessary for VH to occur in schizophrenia. The extent to which people with schizophrenia and VH vary in which factors contribute to VH is also not clear. An additional question is whether some of factors are trait-related (e.g., capacity for more intense imagery; predictive coding impairments), and therefore serve as a necessary setting condition upon which state-related mechanisms impose qualitative changes. The majority of the data reviewed above, however, can be considered to represent trait characteristics, given the focus on patients with VH in general, as opposed to patients experiencing VH during the study session.

**Figure 2:**
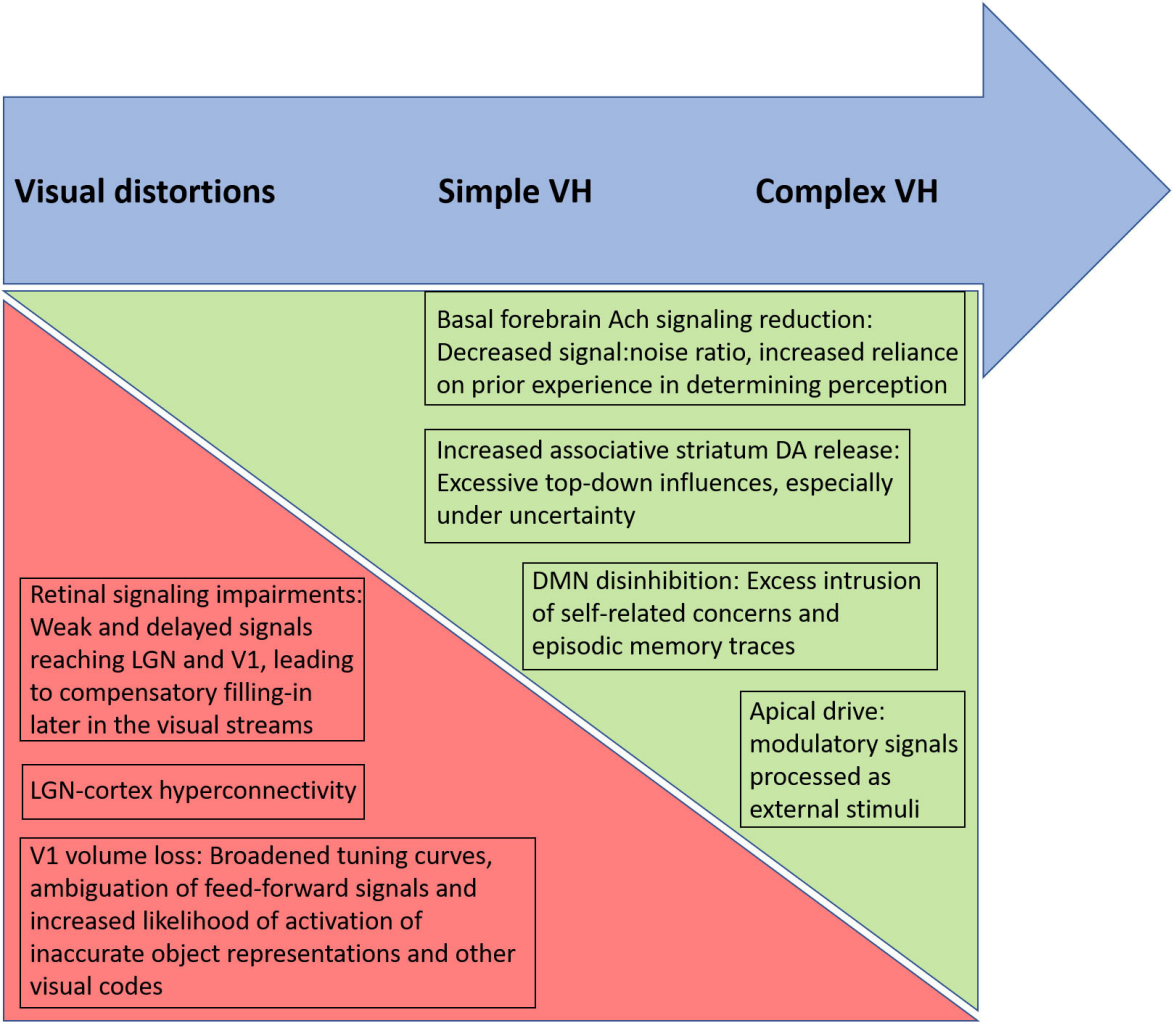
Proposed relative contributions of biological factors, and their psychological consequences, involved in different types of altered visual phenomena in schizophrenia. Arrow at top depicts a continuum from visual distortions to simple visual hallucinations (VH) to complex VH, moving from left to right. Triangles below the top arrow depict hypothesized increasing and decreasing influences across the continuum. The green triangle, becoming larger moving from left to right, represents an increased influence of basal forebrain acetylcholine (Ach) reduction, striatal dopaminergic (DA) increases, default mode network (DMN) disinhibition, and apical drive in generating more complex hallucinatory phenomena in which episodic memory traces and associated emotional factors are thought to be involved. In contrast, the relative contributions of retinal and primary visual cortex changes are hypothesized to be greatest in cases of visual distortions and simple VH, where the anomalous percepts involve filling-in and release phenomena within visual cortex regions. Note that each of these factors involves additional factors (e.g., increased apical drive involves effects of multiple neurotransmitters).

As noted by a reviewer of this paper, the biological and psychological mechanisms discussed above (e.g., disinhibition of DMN, altered predictive coding) are found in many studies of schizophrenia and yet the majority of patients do not have VH and many (1/3) do not have visual distortions. The same issue applies to all work that attempts to identify a mechanism involved in a symptom of schizophrenia, given the large degree of overlap between patient and control samples in studies of potential biological disease markers. For example, with an effect size of *d* = 0.80, generally considered a large effect, there will be 68.9% of overlap between groups, and effect sizes in most PET and MRI studies in schizophrenia are well-below that level (181, 182). Therefore, clarifying which factors and their interactions are involved in VH, *in which people with schizophrenia*, is a critical task for future research. Recognizing that patients may vary in the factors causing their VH, and clarifying which factors and their interactions are involved in VH, *for any single patient*, may therefore be a necessary assessment task required for the effective treatment of VH. In our view, while the state of the evidence does not allow for conclusions to be drawn for all patients at this time, the data do suggest that several candidate mechanisms would be useful to assess within the context of different symptom presentations. For example, in cases where simple VH are dominant, assessment of retinal structure and function, visual evoked potentials, low-level visual functions and occipital lobe structure and functioning may yield useful insights regarding degraded input to V1 and subsequent compensatory filling-in efforts within visual regions, that lead to distortions and simple VH. In cases characterized primarily by complex VH, especially of a threatening or saving (e.g., angelic, salvific) nature, there is likely to be greater involvement of DMN, hippocampus and amygdala activity, and greater contributions of apical drive. We hypothesize that in such cases, reduced cholinergic activity in the basal forebrain and/or elevated striatal DA will cause reduced signal-noise ratios in perception and contribute to an over-reliance on stored representations and emotional factors in driving perception, in a manner analogous to how retinal impairments reduce signal-noise ratio and lead to filling-in phenomena in visual regions in cases of simple VH (see Figure 2).

Testing the views proposed above will involve, first, careful assessment of the nature of the VH in individual patients, and then determining whether the hypothesized impairments can be observed in patients with similar VH characteristics. Using type of VH as the grouping (independent) variable in studies of schizophrenia is not routine, but is probably necessary to answer the questions posed here. It should also be relatively simple to determine if, for example, patients with simple VH demonstrate greater retinal and early visual cortex differences compared to patients without VH, and compared to patients with only complex VH. The latter group, in contrast, should demonstrate more evidence of DMN disinhibition, cholinergic dysfunction, and apical drive, and links between these changes and visual system activity (especially in visual association areas), relative to patients with simple VH. It may be necessary to control for level of auditory hallucinations in studies focused on complex VH since these are likely to share some common mechanisms. It should also be noted that there are no direct tests of apical drive that can be used in human experimental studies; however, extent of apical drive can be computationally modeled from physiological data using information theory metrics (183), and it should be possible to do the same from tests that examine performance as a function of internal influences on perception, such as those showing excess influence of visual imagery on the performance of schizophrenia patients with VH (134–136). Studies examining covariation between aspects of VH and levels of impairments in the mechanisms discussed above would also be useful. And, as noted, investigations of effects of brain stimulation, medications, and other manipulations that affect brain function (e.g., neurofeedback) on aspects of VH could be informative.

The issue of the degree to which VH is a risk factor for schizophrenia also is need of clarification. Clearly, the onset of VH in elderly people is a potential symptom of a condition such as Lewy body dementia or Parkinson's disease. But the predictive validity of VH for a psychotic disorder in a younger person, when eye disease and substance abuse can be ruled out, remains in need of clarification. Also, while some data suggest that the presence of VH indicates a more severe manifestation of schizophrenia, there is much about the clinical significance and course of illness that VH may indicate that is unknown.

Other than knowing that VH, like all psychotic symptoms, can be reduced by dopamine-blocking medications in people with schizophrenia, little is known about their treatment. However, given the high distress level associated with VH, we suggest that further research into effective treatments is warranted. Russo et al. (100) reviewed a range of medication options for VH that are relevant for people with schizophrenia, including anti-serotonergic drugs, acetylcholinesterase inhibitors, typical and atypical (including novel) antipsychotics, and opioid antagonists. Additional treatment directions could involve targeting symptom-related distress (e.g., via cognitive behavior therapy), or the symptom itself via imagery-based interventions (e.g., virtual reality to engage with the content of the VH), trauma-focused treatment to reduce dissociation, and potentially visual remediation and brain stimulation interventions to increase input to cortical regions that are receiving deficient input. Regarding the latter, repetitive transcranial magnetic stimulation (rTMS) over the occipital cortex of a woman with schizophrenia successfully decreased the severity and complexity of her VH (184). RTMS also significantly reduced the auditory and VH in another case study of a man with paranoid schizophrenia (185). Whether this or similar interventions would prove efficacious in randomized controlled trials, however, still needs to be determined.

An issue relevant to both mechanism and treatment is the extent to which even simple VH can represent meaningful concepts and conceptual relationships in people with schizophrenia. It has been noted that visual images can be viewed as propositional representations, wherein basic concepts (e.g., higher/lower, ahead/behind, complete/incomplete, growing/decaying, etc.) are represented, where deformations of images can represent past and future actions, and where images are structured in such a way that other brain modules can decode the meaning they contain—-in short, that visual images are concrete representations that can express complex concepts consistent with a basic “language of thought” that allows for communication between brain modules (167, 186–188). To the extent that this is the case, then even simple VH can be infused with meaning, as demonstrated in Kaminski et al. (15). This suggests that both complex and simple VH may be a useful window into the self.

Finally, there remains the important question of why VH occur less frequently than auditory hallucinations in people with schizophrenia (albeit not as less frequently as thought in the past), whereas the situation is reversed in other neuropsychiatric conditions (e.g., Parkinson's disease). The answer(s) to this question will surely be informative regarding the nature of schizophrenia. One possibility involves findings that some proportion of auditory hallucinations in schizophrenia involve a weakened corollary discharge signal, leading to failure to recognize that subvocal speech activity is internally generated (189–193). It may be the case that there is no parallel in the visual domain to this self-generated or motor program-linked hallucination mechanism (i.e., one is far less likely to motorically generate a visual stimulus than an auditory stimulus), and therefore a weakened efference copy mechanism would be expected to lead to a higher rate of auditory vs. visual hallucinations. Conversely, the visual system mechanisms we discussed above may be more operative in neurodegenerative disorders, and more prominent in these conditions relative to the speech-language center dysconnectivity that has been observed in schizophrenia.

## Summary

Visual hallucinations in schizophrenia are more common than previously thought. They can be viewed as a severe manifestation of visual system changes that occur on a continuum of severity from visual distortions to hallucinations. Several candidate factors for involvement in the genesis of these changes have been proposed and studied, including retinal and visual cortex volume loss and functional impairments, disinhibition of the DMN due to cholinergic and thalamic functional changes, excessive weighting of priors relative to sensory input related to DA (but also Ach and glutamatergic) alterations, excessive apical drive, and hyperconnectivity between limbic and hippocampal regions and visual areas. In general, however, much remains unknown about the causes of VH in schizophrenia, the mapping between contributions of the proposed mechanisms and VH in individual patients, and the clinical significance of VH in terms of treatment and prognosis. One benefit of further research into these questions would be improved understanding of schizophrenia as a neuropsychiatric disorder, including its similarities and differences with other conditions with visual manifestations, such as Parkinson's disease and Lewy body dementia. An intriguing possibility is, to the extent that the content of VH reflects core vulnerabilities of the self (e.g., security/danger concerns), the content of VH can be used to explore and modify dysfunctional cognitive schemata and/or to generate insight into interpersonal and existential issues in the service of behavior change [e.g., (15, 194)].

## Author Contributions

SS conceived of the manuscript. SS and AL conducted the literature review and wrote the sections of the manuscript. All authors contributed to the article and approved the submitted version.

## Conflict of Interest

The authors declare that the research was conducted in the absence of any commercial or financial relationships that could be construed as a potential conflict of interest.

## Footnotes

^1^A rate of VH of 62% was reported in a study of schizophrenia patients in Saudi Arabia (11). As noted by the authors of this study, cultural and religious factors make it difficult to compare these data to rates of VH in countries such as the US and UK.
